# Developing a theory of change to guide the design and implementation of a caregiver-centric support service

**DOI:** 10.1186/s12913-024-11931-y

**Published:** 2024-12-18

**Authors:** Ling Ting Wu, George Frederick Glass, Esther Yin Hui Chew, Emmalene Joo Yong Ng, Ee Yuee Chan

**Affiliations:** 1 Nursing Implementation, Translation and Research Office (NITRO), Tan Tock Seng Hospital, Nursing Service, Annex 1, Level 1, 11 Jln Tan Tock Seng, Singapore, 308443 Singapore; 2 Alice Lee Center of Nursing Studies, National University of Singapore, Singapore, 119077 Singapore

**Keywords:** Theory of change, Implementation, Caregivers, Older persons, Caregiver support programme, Carer stress, Caregiver burden

## Abstract

**Background:**

Set against the backdrop of a rapidly ageing population and growing emphasis on the importance of ageing-in-place, family members often assume the role of a caregiver. Navigating through a complex healthcare system while simultaneously juggling the daily care needs of their care-recipients, caregivers often become worn out by the intense caregiver stress and burden, neglecting their own well-being. This translates to adverse health and economic outcomes such as prolonged hospital stays and increased nursing home placement of care-recipients. Seeking to better support caregivers, we developed a theory of change to guide the design and implementation of a caregiver support programme – Project Carer Matters.

**Methods:**

We applied theory of change methodology to explain how the Project’s interventions were hypothesised to lead to their identified short-to-long term goals, drawing on a causal analysis based on available evidence. The theory of change was developed with insights garnered from previous research studies conducted on caregiver stress, stakeholder engagement sessions and multiple dialogues with clinical experts and hospital leaders.

**Results:**

Our final theory of change is the result of the evaluation of the Project in its pilot phase. It is populated with the resources, activities and short-to-long term outcomes that can be attributed or linked to the Project. Multiple meetings and discussion with stakeholders over the pilot prompted frequent practice of the Plan, Do, Study, Act model to refine the ongoing implementation process and the theory of change itself.

**Conclusions:**

A theory of change is essential in guiding the design, implementation and evaluation of a complex health care intervention such as Project Carer Matters. The development of the theory of change is a journey and not a resultant product. This journey has also led us to learn that 1) a theory of change needs to be dynamic and ever evolving with time and context, 2) the perspectives of relevant stakeholders need to be included in this process to ensure the feasibility and sustainability of the project in the long run and 3) frequent stakeholder engagements are essential in enabling the implementation team to fine-tune the Project in an effective manner.

**Trial registration:**

ClinicalTrials.gov, NCT05205135, registered on 24/01/2022.

**Supplementary Information:**

The online version contains supplementary material available at 10.1186/s12913-024-11931-y.

## Background

The global population of individuals aged 65 and older is projected to double, growing from 761 million in 2021 to 1.6 billion (one in six people) by 2050, and further increasing to 20.7% of the population by 2074 [1–3]. This phenomenon is acutely experienced in Singapore, a developed city-state wherein 25% of its population is projected to be 65 years or older in 2030 [4, 5]. Furthermore, Singapore’s family sizes are projected to shrink, with a decline in the old-age support ratio from 4.8 today to 2.7 in the coming years [6]. Rapidly ageing populations lead to an increasing demand for caregiving, a responsibility often taken on by spouses, siblings, or adult children [7]. To address the rapidly ageing population and associated caregiving demands, more societies like Singapore are pivoting towards a population-focused approach to better enable home-based care for older persons to be cared through improved community-based and home care support, termed “ageing-in-pace” [8]. This approach aligns with traditional Singaporean-accepted values of filial piety, emphasising family members' care and support for their elderly relatives, simultaneously optimising national resources by reducing the need for non-essential institutionalised care, thereby resulting in lower costs and greater value [8].

However, this model often leads to a heavy reliance on family caregivers to care for and help the older persons age-in-place and thrive in the community [9]. Furthermore, the combination of smaller average household sizes in Singapore and the increasing demands of the 'sandwiched generation' propels the need for adult children to perform multiple caregiving roles, with caregiving demand expected to intensify in the near future as Singapore’s population continues to age rapidly [10]. To meet the needs of a greying population and aforementioned increasing caregiving demands on informal caregivers, healthcare systems have gradually recognised the importance of, and transitioned towards adopting a person-centred approach in the care of older persons – the gold standard for aged care [10], which involves fostering a close partnership with patients and their family caregivers to customise care to the patients’ life circumstances, clinical needs, personal preferences and offer skills, knowledge and access that help optimise the patients’ lives [11]. This is essential as person-centred care has been proven to improve clinical and patient outcomes [11, 12]. However, in the process of prioritising patients’ needs and goals in a person-centred care model, caregivers and their well-being are often neglected.

Caregiving is a complex and multidimensional process, requiring family caregivers to not only help with the daily care needs of their care-recipients, but also having to navigate the financial responsibilities of care and address any behavioural issues that may arise from their care-recipients [13, 14]. However, family caregivers in Singapore often enter the role without possessing sufficient knowledge, support, skills, and competencies to provide optimal care for their family members/relatives, particularly when adjusting to the care-recipient’s care needs following a hospital episode or a health-changing event [15–17]. Many family caregivers find the transition and adjustment to the responsibilities of care at home frustrating, stressful and burdensome [18, 19]. In particular, navigation of the complex social and healthcare systems takes a toll on the caregivers [17, 19], with one in three caregivers of older persons at risk of anxiety, depression, and a poorer quality of life due to the highly intensive caregiving duties family caregivers have to bear [14, 20]. The stress and burden experienced subsequently affect caregivers’ ability to care for their family members/relatives in a sustainable manner, which may translate to adverse health outcomes to the care-recipients and raised healthcare costs. These include prolonged hospitals stays, increased hospital re-admissions, nursing home placements and frequent visits to the general practitioners or emergency department [20–23]. Hence, there is a greater public health need for proper preparation, empowerment of and support for family caregivers in Singapore to ensure that they can thrive while caring for their care-recipients, and that their responsibilities are sustainable.

To help meet these challenges, we sought to plan and deliver a caregiver support programme to help caregivers better manage the transition from hospital to home with their family members/relatives – Project Carer Matters. However, as demonstrated above, the multifactorial stressors faced by caregivers require a multifaceted suite of solutions in Project Carer Matters to effectively reduce caregiver stress. This reflects a need for a complex intervention with multiple components, settings and flexibility in delivery. Such a programme had not been explored much in Singapore, which meant that there is a need to evaluate if the interventions led to their intended outcomes de novo [24].

To ensure Project Carer Matters met the needs of caregivers, it was apparent that we needed a clear theory base to underpin it against, with a clear mapping of the causality between the interventions and their hypothesised goal of reducing caregiver stress [24]. This led to us to build a theoretical framework that could allow us to better plan, develop and implement Project Carer Matters, and also to evaluate if the hypothesised changes occurred and adjust the theoretical framework accordingly.

### Defining the theoretical framework

Bearing in mind the multifactorial stressors of caregivers and the subsequent multifactorial interventions needed, a theoretical framework would greatly support the development and subsequent evaluation of Project Carer Matters. This would allow a better capture of the potential interactions between the planned interventions and how they fit into the context of caregiver support whether within their hospital stay or without, after they have been discharged into the community [25].

However, as we were unsure of their potential interactions on the caregivers as they would be developed and introduced to caregivers for the first time, we sought to map our theorised framework of solutions and how they could help as a logic model.

**Fig. 1:**
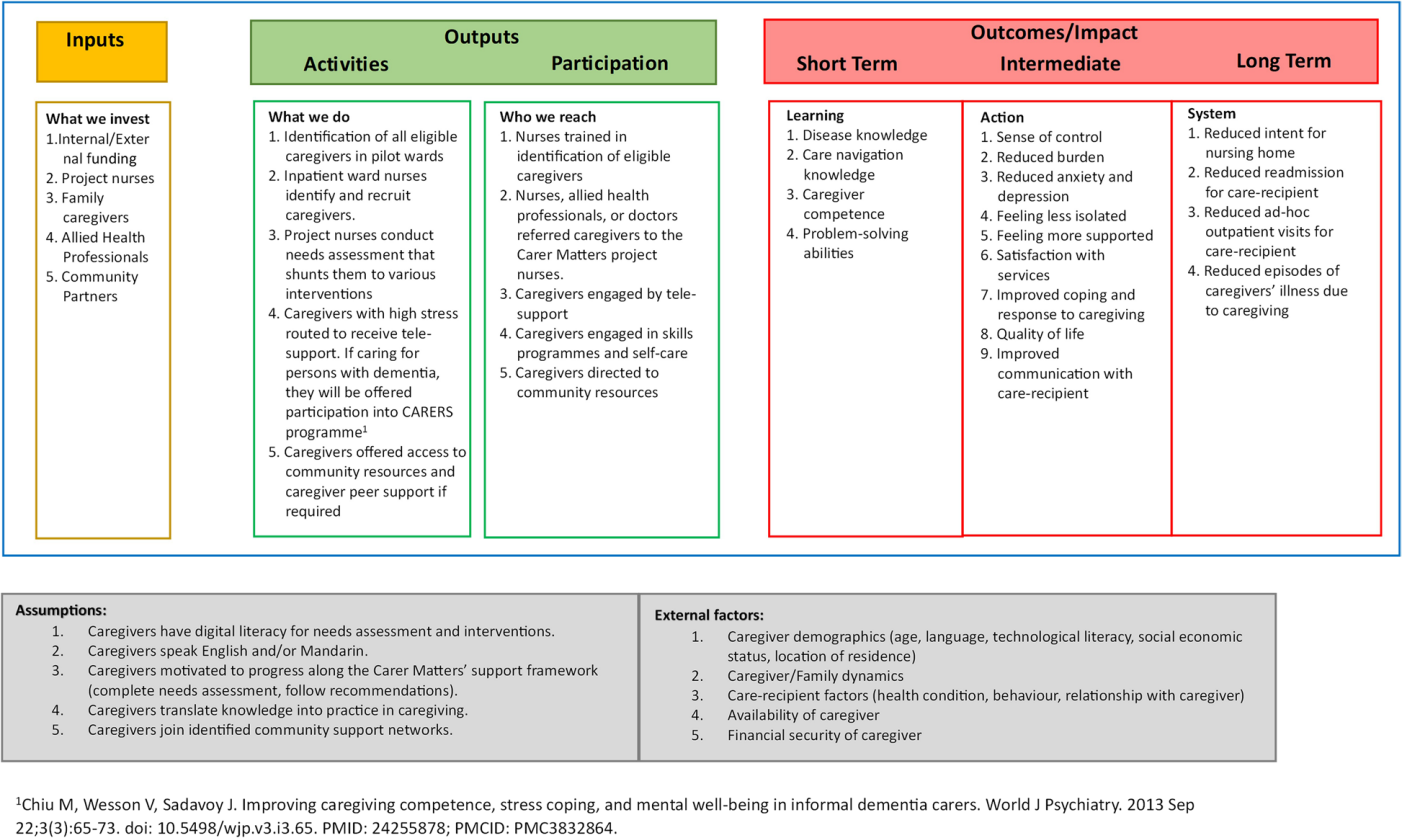
Project Carer Matters initial logic model

A logic model would be a useful first-step towards building our theory as it allowed a visual representation of the underlying rationale to guide our interventions, explicitly demonstrating ‘what works for whom’ in a linear flow stretching from the inputs provided to the targeted outputs [25–27]. This let us better map and identify the best-suited interventions as conceptualized through our earlier work [20, 28] and see how they could contribute to our desired outcomes. Our model was built after the final goals were decided through engagement sessions with stakeholders (Fig. 1), depicting the relationship between the set of inputs and outputs of Project Carer Matters and its desired short- and long-term outcomes.

The initial logic model had to be adapted into a more appropriate framework for Project Carer Matters to capture the multifaceted nature of the programme’s interventions, leading to the development of a theory of change for Project Carer Matters. This represents a hypothesis about the way a programme brings about its effects [29]. The theory of change concept has gained visibility in recent years as an alternative method to conceptualise and evaluate public health programmes, delivering upstream interventions that are intended to lead to short-term, intermediate and long-term outcomes [30]. The theory of change offers a pragmatic conceptual framework that focuses on mapping out the rationale of how a programme works and how it achieves its goals in reality as compared to traditional experiments conducted in artificially-controlled settings [31, 32]. It also outlines, often visually, how an intervention achieves long-term outcomes through specific intermediate outcomes, often summarised in ‘pathways of change’ [33].

In the context of Project Carer Matters, we initially employed a logic model to outline our hypothesis—that interventions such as providing caregiver support, offering caregiving courses, and fostering community support and connections will reduce caregiver stress, enhance mastery and self-care, and ultimately, result in a long-term reduction of the burden on the healthcare system. We sought to apply our evaluation of Project Carer Matters, and subsequent understanding of the causal linkages between the interventions and outcomes, to flesh out a Theory of Change theoretical framework.

## Methods

### Study design

We sought to establish a theoretical framework to guide the development of Project Carer Matters and to subsequently explain how Project Carer Matters interventions are hypothesised to lead to the short-, intermediate-, and long-term goals identified, drawing on a causal analysis based on available evidence. The Project was piloted in a tertiary hospital in Singapore, from January 2020 to December 2021. The pilot phase of the Project was evaluated using the Reach, Effectiveness, Adoption, Implementation and Maintenance (REAIM) framework [34] leading to the development of a theory of change as reported below.

### Development of theory of change

The theory of change was developed with insights garnered from (1) literature review (2) previous research studies conducted on caregiver stress and mastery during the post-discharge transitory phase, as the patients transition from hospital to home [17, 20, 27, 35], (3) stakeholder engagement sessions and (4) multiple dialogues with clinical experts and hospital leaders. It was also adjusted based on the modifications to the interventions carried out by the implementation team over the pilot period, conducted over iterative rounds of the Plan, Do, Study, Act (PDSA) model [36].

Led by the Project’s lead, the theory of change was developed by the evaluation team, which included an implementation science expert, a group of nurse researchers, supported by the implementation team who delivered the interventions, consisting of project nurses, front-line nurses and operational staff.

In this paper, we developed and reported the theory of change according to the checklist for reporting Theory of Change in Public Health Intervention by Breuer et al. (2016). Breuer’s checklist covers five domains to ensure that a theory of change is clearly reported [30]. These include (1) defining theory of change; (2) describing the development process of theory of change; (3) depicting the theory of change in a diagrammatic form; (4) mapping the process of intervention development and (5) describing the use of theory of change in evaluation. (Refer to supplementary Table 1 for further breakdown of the five domains).

The final goals of Project Carer Matters were first set out together with stakeholders including clinical experts and hospital leaders. We considered what could be directly achieved through the Project and what was beyond its sphere of influence, as some of the long-term goals may not be directly achievable. In particular, these long-term goals remain dependent on a wide variety of conditions and factors in the healthcare ecosystem that are beyond the control of the Project, such as the caregivers’ own socioeconomic background and extent of family and social support [35]. With the final goals decided upon, we worked backwards to consider achievable intermediate outcomes that could lead to the final goals. Subsequently, activities were planned to link the interventions to the relevant short, intermediate and long-term outcomes. The rationale for these interventions and the primary assumptions as to how these interventions could translate to the hypothesised outcomes were also included.

With the logic model established, we were able to refer to it as we planned the delivery of our interventions. For example, recognising the importance of the inpatient nurses in identifying eligible caregivers for support, the implementation team embarked on the recruitment and support of peer champion nurses within the wards, who helped encourage and support the on-the-ground activity of caregiver screening. This in turn ensured as many caregivers were identified to receive the needed support. Details on the interventions and their mechanisms, as developed through our framework, are found in our earlier publications [24, 34].

**Fig. 2:**
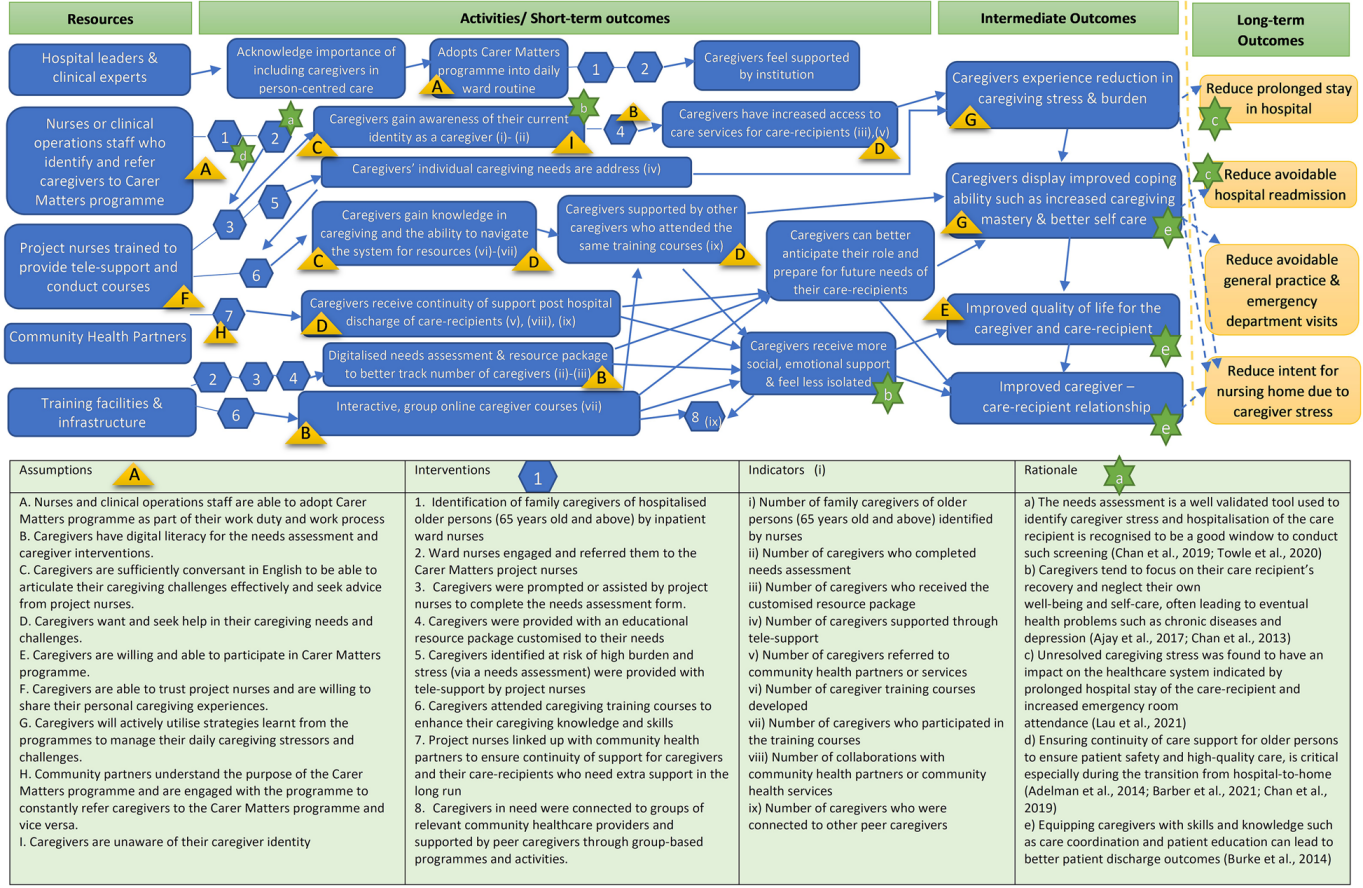
Project Carer Matters theory of change model

However, due to the complexity of the programme, we recognised that the logic model has its limitations in defining assumptions and the necessary preconditions to link the interim outcomes needed to reach the long-term goals in a sequential format [37]. While the initial logic model provided a useful framework to illustrate the linkages and causal relationships between the set of inputs, activities and the organisation’s desired outcomes, it overlooked other factors such as contextual variations during the implementation phase that contribute to the observed outcomes of the Project, which is a complex adaptive intervention [31, 38, 39]. A theory of change model was subsequently developed to better depict the rationale and causal links between the Project’s interventions and the long-term outcomes over a sustained period of time (Fig. 2).

To better address these gaps, we applied an Implementation Science strategy to test our framework, to understand the barriers and facilitators to the uptake of our planned solutions across the different contextual levels such as healthcare providers and caregivers [40]. To achieve this, we adopted the Reach, Effectiveness, Adoption, Implementation and Maintenance (REAIM) framework, to examine the impact of the Project and changes made. Our findings are reported in our earlier publications [24, 34, 41].

We integrated the findings from this study in our theoretical framework, fleshing out the interactions across the interventions and how they contributed towards the caregivers feeling better supported and less stressed. This led to our theory of change model, reflecting the rationale and causal links between the Project’s interventions and the long-term outcomes over a sustained period of time (Fig. 2).

## Results

### Refined theory of change

Our refined theory of change as illustrated in Fig. 2, was successfully utilised as a ‘guiding, designing and implementation tool’ for Project Carer Matters. Multiple meetings and interviews were held with caregivers, implementation team, evaluation team, nursing leaders, organisation’s leaders, ward nurses, community healthcare providers. A consensus was reached on the operationalisation of the short-, intermediate-, and long-term goals for the Project, in terms of its indicators and outcomes. Through these meetings, commitment and collaboration amongst the team members were established. Key assumptions and pre-conditions for each outcome and possible indicators were listed and denoted in Fig. 2 to clearly depict the relationship between these factors. Arrows were added to illustrate the process, mechanisms and causal links between the interventions and the short-, intermediate-, and long-term outcomes the team had mapped out.

### Outcomes for Project Carer Matters identified

With the insights garnered from literature reviews, our earlier research on caregiver stress during the patient’s transition from hospital to home [17, 20, 23, 27, 35], and the meetings and dialogues with stakeholders (i.e. caregivers, clinicians, hospital leaders and community leaders), the following long-term goals were established: 1) reduction in prolonged hospital stays; 2) reduction in avoidable hospital readmissions; 3) reduction in avoidable visits to the general practitioners and emergency departments; and 4) reduction in intent for nursing home. During the interviews with stakeholders and multiple meetings conducted with the implementation team, it was acknowledged that these long-term goals cannot be directly achieved through the Project alone, due to the multiple interplaying factors that lay beyond its control. For example, a patient's length of stay in the hospital can be influenced by many factors such as the patient's medical condition, patient’s readiness to return home, caregiver’s work arrangements as well as caregiver’s and patient’s personal or family matters. Hence, while the Project may have intervened, it is hard to directly attribute a reduction in prolonged hospital stays to the Project alone.

Hence, we chose to focus on achievable intermediate outcomes that contributed towards the final goals. Through discussion with stakeholders, it was agreed that considering other preconditions and factors, these intermediate outcomes are achievable in the long run and could play a vital role in bringing the long-term goals to fruition. As shown in Fig. 2, dotted arrows were used to denote the causal links from the intermediate outcomes to the long-term goals, indicating that the intermediate outcomes contribute to, but may not have direct impact on the long-term goals. Likewise, the activities and short-term outcomes were clearly indicated with straight line arrows to indicate direct linkages from the short-term outcomes to intermediate ones. For example, during the interviews with stakeholders, caregivers verbalised that the caregiver training programme enabled them to gain knowledge in caregiving, and they also felt aided by the peer support received from peer caregiver attendees. These interventions have helped them to feel more emotionally supported and cope better as a caregiver [34], achieving the intermediate outcomes outlined in the theory of change. This is illustrated by the straight-line arrows that depict the direct causal linkage between the short and intermediate outcomes (Fig. 2).

### Project Carer Matters interventions

Project Carer Matters is founded on two fundamental tenets: 1) orientating caregiver training towards mastery-building and 2) driving a holistic caregiver-centric approach. It is a caregiver-centric ‘Hospital-to-Home’ project that holistically screens, identifies and provides targeted interventions for caregivers of older persons identified to be at risk of high caregiver burden [24]. In this project, allied health professionals, geriatricians, nurses and social service agencies work together towards improving caregivers’ well-being and their capacity to care for their care-recipients in a sustainable manner [24].

As stated earlier in the paper, the Project’s interventions augment caregivers’ emotional preparedness, mental and psychosocial resilience and drive long-term engagement initiatives that integrate caregivers into an embedded network of community-support services and peers [24]. Additionally, the interventions were designed based on research evidence garnered from studies, which found that caregivers must be better equipped with skills and rendered essential support during their caregiving journey to reduce caregiver anxiety and burden [42]. These mapped interventions were raised for discussion during stakeholder engagement sessions and dialogues with the relevant clinical experts, community partners and hospital leaders for an assessment of their feasibility and overall alignment with the hospital’s organisational directions.

The interventions in the Project were carefully mapped out so that the short and intermediate outcomes could be achieved. The multi-pronged strategy included the following interventions: 1) Identification of family caregivers of hospitalised older persons (65 years old and above) by inpatient ward nurses, 2) Ward nurses engaged and referred family caregivers to the project nurses, 3) Caregivers were prompted to complete the needs assessment form. Caregivers who were unable to complete the form independently were assisted by project nurses, 4) Caregivers were provided with an educational resource package customised to their needs, 5) Caregivers identified at risk of high burden and stress (via a needs assessment [43]) were provided with tele-support by project nurses, 6) Caregivers attended caregiving training courses to enhance their caregiving knowledge and skills, 7) Project nurses linked up with community health partners to ensure continuity of support for caregivers and their care-recipients in the long run, and 8) Caregivers in need were connected to groups of relevant community healthcare providers and supported by peer caregivers through group-based programmes and activities.

These interventions were listed in the theory of change (Fig. 2) and each intervention with its representative number were included in the figure to clearly indicate where each intervention was implemented over the course of the entire project. Straight line arrows were used to depict the linkages between the Project’s resources, activities and relevant outcomes that were directly achievable through these interventions. As illustrated, activities such as acknowledging the importance of caregiver welfare and helping them feel supported aided caregivers to better cope with their caregiving role and improve self-care.

**Fig. 3:**
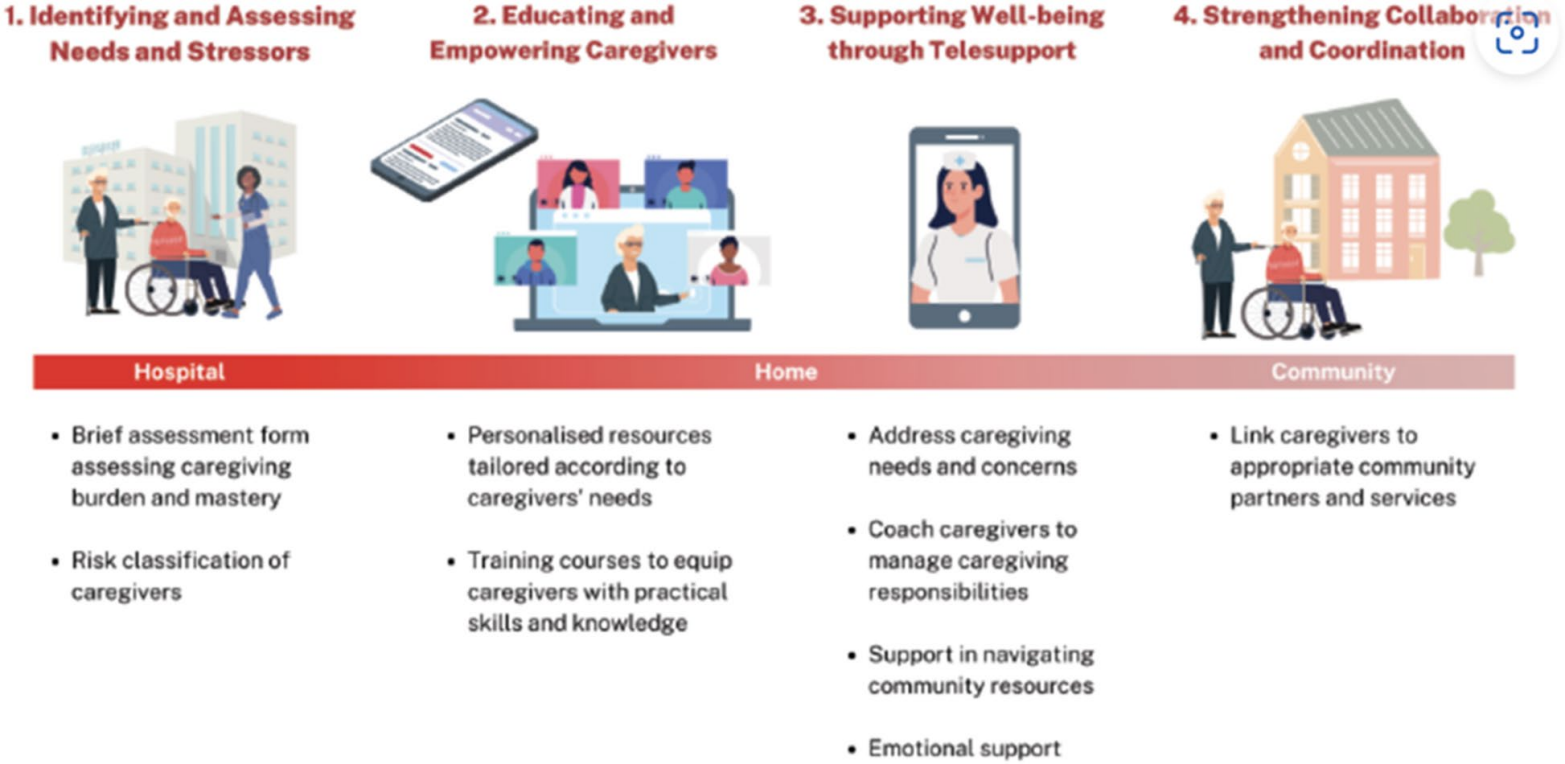
Flow of Project Carer Matters interventions

A more detailed description of the Project’s interventions has also been reported previously [34]. For reference, a diagrammatic flow chart (Fig. 3) of the interventions as well as a table (Table 1) to summarise the caregiver training courses conducted by the Project’s implementation team are included below [10, 24].

**Table 1:** Summary of Project Carer Matters training courses for caregivers

Training Courses	Course Description
CARERS (Coaching, Advocacy, Respite, Education, Relationship. Simulation) Programme [44]	This therapeutic evidence-based group intervention features a unique hands-on simulation exercise in the presence of a simulated patient to practice the application of problem-solving techniques. The course is co-led by two facilitators and held weekly over eight weeks in small groups of four to six participants and is offered to caregivers of persons with dementia
TEACH Programme	This interactive group course aims to build caregiving skills and provide emotional support for family caregivers. Sessions are tailored to central themes of caregiving (changing relationship, community resource navigation, future planning, self-care)
Understanding Dementia	This course helps caregivers understand dementia, the nature of Behavioural and Psychological Symptoms of Dementia (BPSD) and general approaches to challenging behaviours
Problem-solving techniques	This course introduces a five-step problem solving technique adapted from the CARERS programme [44]. This is a group session tailored to help caregivers address practical problems faced [44].
Self-care	This course helps caregivers recognise the importance of self-care and learn practical self-care tips
Caregiving Essentials	This group course helps caregivers understand more about caregiving and provide practical caregiving tips
Public Forums/Seminars	The seminars are designed to provide generic sought-after information for caregivers, such as financial support availability and home safety

### Key assumptions identified by stakeholders

Although the interventions and short- to long-term goals of Project Carer Matters have been carefully planned out through multiple layers of engagement and interviews with stakeholders, the implementation team acknowledged that the Project may not cater to all caregivers. Key assumptions were, therefore, identified to justify how the stated interventions could lead to one or multiple outcomes. For example, due to resource limitations, the Project’s materials were available only in English. This was because English is the de facto language spoken by Singaporeans. However, Singapore is a multilingual society with locals speaking four main languages (English, Mandarin, Malay, and Tamil) as well as many local dialects. Hence, family caregivers who are not sufficiently conversant in English may find it challenging to articulate their caregiving challenges effectively. Further translation and adaptation of the Project’s materials into languages beyond English will therefore be needed for the Project to achieve its aim of benefitting all caregivers in Singapore.

A list of key assumptions highlighted by the stakeholders are clearly listed in the theory of change model (Fig. 2). The key assumptions with their corresponding alphabets are also included in the same figure to clearly indicate where these assumptions were made in the process of crafting the linkages between the interventions and hypothesised outcomes.

### Adaptations made to Project Carer Matters interventions

As guided by the initial theory of change, Project Carer Matters was piloted to evaluate the feasibility and sustainability of the Project. As with many other pilot programmes, the Project faced implementation challenges, which led to the need for programme modifications and further alteration of the theory of change. Multiple meetings were held between the evaluation team, implementation team and inpatient ward nurses. These discussions on implementation challenges elicited frequent practices of the PDSA model to refine the ongoing implementation process as well as the theory of change [36, 43]. During the same period, the evaluation team also conducted semi-structured, individual interviews with 51 stakeholders of the Project including inpatient ward nurses, project nurses, caregivers, community representatives and hospital leaders [34]. These key stakeholders were interviewed on their experiences and perspectives of the potential barriers and facilitators of the implementation of Project Carer Matters, suggestions to streamline its implementation, and its feasibility in achieving its long-term outcomes. Findings from these interviews were shared in our earlier publications [34].

The implementation challenges are listed below, together with the resulting modifications made to the interventions. A consensus on the alterations to the interventions was built through interviews conducted with stakeholders and multiple meetings held with the implementation team. The challenges and adaptations made to the relevant interventions are summarised in Table 2.

**Table 2:** Adaptations made to Project Carer Matters interventions

**Objective **	**Initial intervention **	**Refined intervention **
Increase exposure and publicity of Project Carer Matters to help caregivers better identify their role as a caregiver.	• Inpatient ward nurses approached caregivers during admission.	• Brochures on Project Carer Matters placed in the post-discharge package provided to caregivers and patients. • Large posters on Project Carer Matters placed strategically in ward lifts and on digital information boards. • Project Carer Matters’ microsite set up (Project Carer Matters Webpage) [45]. • Publicity video displayed on social media to increase awareness on Project Carer Matters.
Recruitment of family caregivers	• Inpatient ward nurses identified, engaged, and recruited the caregivers when they visit the hospital.	• Inpatient ward nurses sought caregivers’ agreement to be contacted by project nurses and simultaneously directed caregivers to complete the caregiver assessment via a QR code. • Other clinical staff trained to refer caregivers to the project nurses. • Caregivers scanned Project Carer Matters QR code and completed needs assessment independently. • Caregivers who are unable to complete the needs assessment form independently are assisted by project nurses.
Delivery of caregivers’ training courses	• In-person and on-site training courses (i.e., hands-on simulation).	• Courses conducted online during COVID-19 pandemic. • Caregivers engaged in caregiving simulations via ZOOM platform during the courses. • Project nurses re-enacted scenarios to help Caregivers role-play virtually.
Overlapping services with community healthcare providers who offer support to caregivers	• Project nurses confirmed with caregivers if they received follow-ups from other hospitals or community services.	• Project nurses met up with community healthcare providers to clarify on the different roles and responsibilities of Project Carer Matters. • Project nurses clarified with community healthcare providers about the time period Project Carer Matters would support caregivers. • Collaborated with community healthcare providers to follow up on caregivers’ needs in the community.

#### Lack of caregiver self-identification

In a collectivistic society like Singapore that emphasises notions of filial piety and familism [35, 46], many family caregivers do not identify themselves as caregivers. They perceive their caregiving duties as an extension of their role as their care-recipient’s child or spouse, often putting emphasis on their care-recipient’s need while neglecting their own [35]. Others did not perceive themselves as being a caregiver as they often associate caregiving responsibilities to the tasks performed by hired lived-in domestic helpers owing to the similarities in the physical nature of the tasks [34]. Additionally, some family caregivers did not recognise the need for additional caregiver support from the Project, as they felt that they were self-sufficient and well-equipped to care for their family members/relatives. The lack of awareness and recognition for the need for assistance in caregiving posed as barriers to caregivers’ participation in the Project.

In order to increase awareness and enable caregivers to self-identify their caregiving role and potential risk of having caregiving burden, stakeholders have identified the need for Project Carer Matters to improve its publicity and outreach efforts. A wide variety of media and platforms were launched throughout the hospital to alleviate the pressing challenge of caregivers’ lack of awareness of the Project. Such adaptations to the publicity efforts aimed to help increase the Project’s visibility, boost family caregivers’ ability in self-identifying as a caregiver and motivation to seek support and assistance in relieving their caregiving burden. The refined publicity or marketing efforts included providing publicity brochures in each of the caregiver’s post-discharge package; placing large posters strategically in ward lifts and on digital information boards; creating a microsite of the Project that was hosted on the hospital’s website [45] and displaying a publicity video showcasing the Project on social media (e.g., YouTube) to increase its outreach and visibility.

#### Challenges in recruiting family caregivers

The arrival of COVID-19 led to the swift implementation of necessary safe distancing measures to stem the spread of the pandemic. Despite attempts to integrate Project Carer Matters’ recruitment efforts into the ward nurses’ workflows to ensure the referral process was as effortless and seamless as possible, other more pressing demands relating to patient care were inevitably prioritised over the recruitment of caregivers. The busy nature of the inpatient wards and restrictive visiting hours and attendant reduced ward nurses’ engagement with the family caregivers, made worse by the COVID-19 pandemic, further dampened caregiver recruitment for the Project. Consequentially, as caregivers’ entry into Project Carer Matters was entirely dependent on their identification and recruitment by the inpatient ward nurses, the Project’s recruitment efforts for both low- and at-risk caregivers were hampered as the nurses had to place more focus on their routine patient care.

Modifications were, therefore, made to the caregiver recruitment process to facilitate the Project’s recruitment and lighten the inpatient ward’s workload simultaneously; instead of having inpatient ward nurses take time out of their work schedules to engage, recruit and encourage the family caregivers to join the Project, they only had to seek family caregivers’ agreement to be contacted by the project nurses. The project nurses would then explain the Project’s details to the caregivers and, through prompting and active engagement, facilitate the caregivers’ completion of the caregiver needs assessment. Other clinical support staff working within the ward-setting with frequent contact and touchpoints with the family caregivers (e.g., patient service associates), were similarly trained to refer and direct eligible family caregivers to the project nurses for follow-up.

Additionally, with increased publicity and digitalisation – as discussed in point (1) – caregivers need not be recruited by nurses or other healthcare staff but join the Project independently through scanning of the QR codes that can be found on the posters and digital signboards publicly visible throughout the hospital grounds, thereby improving the sustainability of Project Carer Matters and reducing the strain of recruitment on healthcare staff.

#### Challenges in the delivery of caregivers training course in Project Carer Matters

Amongst the caregiver training courses, CARERS (refer to Table 1) is a signature course offered to caregivers of persons with dementia [47]. It is an evidence-based caregiver training that was originally conceptualised and developed in Canada [44]. During the COVID-19 outbreak, the team subsequently prototyped a virtual version of the CARERS programme to allow learners to attend off-site, and better equip family caregivers in their caregiving responsibilities for their family members/relatives [5]. The CARERS programme was valued by the implementation team as it allowed them to address local caregiver needs, founded on principles of problem-solving techniques, adult-learning and experiential learning, which equipped caregivers to better manage their caregiving needs.

The CARERS programme comprises of a simulation exercise that provides caregivers an opportunity to engage in role-play, which revisits their caregiving challenges and scenarios that surfaced in the home-setting. However, during the pilot testing of the project, many caregivers highlighted that it was challenging to feel engaged in the simulation exercise and role-play virtually in front of a screen [34]. As a result of this technological drawback, project nurses made efforts to re-enact the different scenarios and ensured that caregivers were clear of the simulation objectives before guiding them to engage in a ‘virtual role-play’. This helped to minimise the lack of non-verbal cues, which are inherent in online courses.

#### Overlapping services for caregivers in the hospital/community

Project Carer Matters aimed to educate, equip, and empower caregivers through tele-support and programmes to better support care delivery for enhanced seamless hospital-to-home post-discharge transition. Such interventions overlapped with some of the roles and responsibilities of the community healthcare providers, which involved caregivers when patients transited from hospital to home. Some caregiver training courses were also available at the community level to help caregivers better understand their family members/relatives’ medical condition and were perceived to have overlapping content with project’s training courses.

Indeed, recognising that there were pre-existing overlaps between the role of project nurses and community healthcare providers, the project nurses constantly liaised with community partners to strengthen collaborations. Through frequent meet-ups and dialogues, the project nurses were able to clarify on the different services and roles and responsibilities of the Project and collaborate with the care providers on how to collectively better support caregivers. Additionally, caregivers were referred to the community partners for further support on their identified caregiving needs. Considerable coordination and collaboration with other community healthcare providers is key to establishing Project Carer Matters’ niche spot in the ecosystem. That way, together with other caregiver-facing healthcare providers, the Project is uniquely positioned to address caregivers’ needs.

With changes made to the Project interventions as detailed above and in Table 2, the theory of change has been refined from one that is suited for inpatient recruitment as well as active identification of caregivers in the wards, to one that allowed multiple avenues of publicity that helped increase awareness of caregivers. In-person caregiver training courses were converted to virtual courses to increase flexibility while increased community partnerships offer better caregiver support that aid families/relatives to truly ‘age in place’. The refined activities were thought to be more sustainable, leading to a theoretical framework that helps to streamline the hospital-to-home caregiver support model that will bring about the potential long-term impact on the healthcare system.

## Discussion

The theory of change is increasingly used to develop and evaluate complex public health interventions, boosting the chances of such interventions being ultimately scalable, sustainable, and effective [48]. This article sets out the development of the theory of change for Project Carer Matters and how it illuminates the linkages and causal relationship between the set of inputs, activities, and the organisation’s desired outcomes for the family caregiver support project, specifically highlighting the rationale behind the interventions. To achieve this, a participative process was intrinsic to the building of the initial logic model and subsequently, the theory of change model. A combination of insights gathered from 1) literature reviews; 2) prior research studies conducted on caregiver stress and mastery during the post-discharge hospital-to-home transitory phase; 3) stakeholder engagement sessions and 4) multiple dialogues with clinical experts and hospital leaders where expertise was provided, highlighted the importance of caring for family caregivers so that they can better care for their older care-recipients. Given that the existing care model in the hospital and the community are patient-centric in nature, Project Carer Matters was implemented as a caregiver-centric project that aimed to address the current gaps in caregiver support. This has promoted a paradigm shift from a patient-centric care to a care model that supports both the patient and caregiver.

However, in the process of developing a theory of change and pilot testing the Project, it is clear that while the interventions were initially planned out through theory, frequent amendments and adaptations are required to anchor the interventions’ feasibility in the healthcare system’s fast-paced environment. From a conceptual standpoint, the Project’s lead and implementation team were clear of the Project’s goals, the desired short- and long-term outcomes as well as the interventions required to manifest the change. However, it takes continuous collaborative efforts from stakeholders, healthcare leaders, implementers, collaborative partners, as well as the caregivers themselves to initiate changes, as illustrated in our theory of change model. As suggested by Vogel (2012), a theory of change can enhance the impact of interventions as they stimulate practitioners to include the perspectives of various stakeholders when theorising how the overall intended outcomes could be best achieved by a complex intervention [48–51].

In this article, the theory of change has evolved and refined with time, adjusting throughout the implementation and evaluation of the intervention, thus, enabling a continuous process of reflection on how change occurs [52]. While the initial theory of change was used to guide the design and implementation of the Project in its pilot phase, evaluation of the Project’s feasibility led to further modification of the theory of change. Stakeholders’ involvement in the development of the theory of change also created a sense of ownership and buy-ins from relevant stakeholders [53]. Ideally, all stakeholders should have ownership over the theory of change [53]. However, due to the complexity of the Project, which cuts across various care settings from hospital to home, this is often difficult to execute in practice. Hence, Project Carer Matters focused on implementation ownership, whereby the implementation team assisted in fact-checking and confirming the final theory of change. The final theory of change could also be potentially used as a conceptual tool to guide the implementation of Project Carer Matters and bring about the hypothesised changes in the future, such as aligning its scale-up processes with contextual changes and stakeholder involvement, potentially increasing the likelihood of its successful expansion [28]. According to Breuer et al. (2016)‘s checklist for reporting Theory of Change in Public Health Intervention, Domain 5 includes the use of the theory of change in project evaluation. In this report, our final theory of change has yet to be used for further evaluation of the Project. Given that the theory of change evolves alongside the interventions of Project Carer Matters, it is suggested that the theory of change be used to guide future large-scale implementation and evaluation of the Project specifically through a quantitative measurement of the outcome indicators in relation to its interventions.

Family caregivers are the main beneficiaries of Project Carer Matters, wherein they would be trained to possess skills and knowledge to care for their family members/relatives and ensure self-care in the process. They would also learn problem-solving techniques to overcome unforeseen future caregiving obstacles, and enhance their capacity to acknowledge and address their own caregiving needs and manage their caregiving burden and the associated negative emotions [5]. Ultimately, when caregivers cope well with their caregiving tasks with decreased distress, they will enjoy optimal health as they continue to provide sustained and effective care to their family members/relatives [35]. However, through the process of developing the theory of change for Project Carer Matters, it became clear to the evaluation team that the healthcare interventions could neither be solely patient-centric nor caregiver-centric. Even though the caregiver and care-recipient are two different individuals with different needs, their social and emotional well-being are often intricately intertwined [54]. Programme overlaps that arose between the Project and community healthcare providers were likely because the Project’s interventions were designed to be more caregiver-centric while the existing services offered by the community healthcare providers were designed to be more patient-centric. In the process of implementing the Project’s interventions and collaborating with the community healthcare providers, the importance and necessity to perceive the patient-caregiver dyad as one unit – addressing the needs of both the patient and their caregiver together – was realised. As evidenced in a previous study conducted on heart failure patients and their caregivers, it was found that patients' symptom burden led to caregivers' depressive symptoms while caregivers' caregiving burden contributed to patients' depressive symptoms. These interdependent relationships suggest that dyadic interventions focused on reducing burden and perceived stress may be beneficial for relieving depressive symptoms in patient–caregiver dyads [55]. Future studies could consider the value of dyadic caregiver-patient interventions, targeting both the patient and their caregiver when transiting from hospital to home, and subsequently into the community.

From hospitalisation to post-patient discharge, Project Carer Matters was initially designed to support caregivers in a linear, stepwise manner, starting from screening and identifying caregivers at the point of patient admission. The identified at-risk caregivers would subsequently receive interventions that would augment their caregiving skills, knowledge, health, sense of capability and mastery and subsequently improve their kinship with their family members/relatives [24]. However, in reality, caregivers may not participate in or complete all of the Project’s interventions. Caregivers’ needs are dynamic and ever-evolving throughout the course of their care-recipient’s illness and their own lifetime; this means that caregivers would require different types of services and support at different points in their caregiving journey. Additionally, developing a long-term relationship with caregivers would be necessary to facilitate continuous engagement rather than one-off interactions that tend to be more transactional in nature. In essence, the theory of change model illustrates how caregivers may embark on different causal pathways as part of the Project’s interventions, which eventually leads to the mapped varied short- to long-term outcomes. The theory of change further emphasises that there is no ‘one size fit all’ programme for caregivers who are diverse individuals and interventions should be tailored according to caregiver’s heterogenous needs and coping ability.

## Conclusions

In conclusion, a theory of change is essential in guiding the design, implementation and evaluation of a complex health care intervention such as Project Carer Matters. The final theory of change potentially serves as a conceptual guide to envisioning how Carer Matters’ interventions can continue to impact changes through the mapped short- to long-term outcomes. Essentially, the journey of developing the final theory of change has led to a few salient learning points. Firstly, the process of developing a theory of change needs to be one that is dynamic and constantly evolving with time and context, aligned with the rapid changes in health landscape. Secondly, the theory of change should also consider insights from multiple stakeholders including hospital leaders, clinical experts, implementers, community partners, caregivers and their care-recipients to ensure the feasibility and sustainability of the Project in the long run. Finally, conducting frequent stakeholder engagements is essential to the development of the theory of change as their valuable inputs have helped the implementation team to fine-tune the Project in an effective manner that better benefits the caregivers and care-recipients. The Theory of Change itself enhances the impact of our interventions as it drives programme developers to examine the perspectives, needs and priorities of all impacted stakeholders when examining how a complex multi-component intervention can ‘hit the mark’ of all intended outcomes. While the Project’s interventions were planned to be caregiver-centric, some of the challenges encountered during its implementation phase points us to an important shift towards acknowledging the necessity of mapping and implementing dyadic interventions that encompass the support for both the caregivers and their care-recipients.

## Supplementary Information

Supplementary Material 1.

## Data Availability

The datasets generated and/or analysed during the current study are not publicly available as the ethical approval for the study does not permit data sharing, but are available from the corresponding author on reasonable request.
