# Supplementary material for: Developing a theory of change to guide the design and implementation of a caregiver-centric support service
Source: BMC Health Serv Res. 2024 Dec 18;24:1620. doi: 10.1186/s12913-024-11931-y (PMC11658152; doi:10.1186/s12913-024-11931-y)
Supplement: Supplementary file 1 — Supplementary Material 1. [file 12913_2024_11931_MOESM1_ESM.pdf]

Supplementary Material

Table 1: Checklist on reporting theory of change in public health interventions (Breuer et al., 2016)

Items	
1. Is the theory of change approach defined?	
a. Is a definition of the theory of change given by the authors?	Page 5, Para 1-2
b. Do the authors explain their reasons for using a theory of change approach?	Page 7 - 8
2. Is the theory of change development process described?	
a. Are the methods used to develop the theory of change, such as stakeholder meetings and interviews, document reviews, programme observation, existing conceptual frameworks or published research, described?	Page 6 - 8
b. Where stakeholders are involved, is it clear how many stakeholders participated, what their role is in relation to the intervention, how they were consulted (e.g. number of interviews, focus groups, theory of change workshops) and the extent to which the consultations were participatory?	Page 8 -13
c. Is the method used to compile the data into a theory of change described? (including how disagreements between stakeholders were resolved)	Page 13 -19
d. Is the extent to which stakeholders were able to validate the resultant theory of change and were owners of the final product described?	Page 25 para 1
3. Is the resultant theory of change (or a summary thereof) depicted in a diagrammatic form and does it include?	
a. The long-term outcome or impact of the intervention	Figure 2
b. The anticipated short- and medium-term outcomes and the process of change	Figure 2
c. The intervention components which happen at different stages of the pathway	Figure 2
d. The context of the intervention	Figure 2
e. Assumptions about how change would occur	Figure 2

f. Additional theory of change elements such as indicators, supporting research evidence, beneficiaries, actors in the context, sphere of influence and timelines where relevant.	Figure 2
4. <u>Is the process of intervention development from the theory of change described?</u><br><br>a. Are the methods of how interventions were refined from the theory of change to something which can be implemented described? (For example, further stakeholder workshops, interviews, systematic literature reviews)	 <br><br>Page 13 - 19
5. Is the way in which the theory of change was used to develop and implement the evaluation described?<br><br>a. Are evaluation research questions generated from the theory of change?<br><br>b. Is the role of theory of change in the design, plan or conduct of the evaluation clear?<br><br>c. Does the paper describe the extent to which the key elements described in the theory of change were measured in the evaluation (i.e. impact, short and medium term outcomes and the process of change, context, assumptions and the intervention)?<br><br>d. Does the paper describe whether and how process indicators were used to improve the quality of the intervention?<br><br>e. Is the role of the theory of change in the analysis of the results of the evaluation clear?<br><br>f. Is the role of theory of change in the interpretation of the results of the evaluation described? (including the breakdown of programme theory, unanticipated outcomes and causation including the strength and direction of causal relationships)	Project Carer Matters was piloted in 2020-2021 and the evaluation of the pilot phase has been reported in a previous publication (Chan et al., 2022)<br>Our final theory of change reported in this article has evolved through time due to the adaptations made to Carer Matters interventions and the context during which the project was piloted.<br>Further use of the theory of change to evaluate Project Carer Matters in a larger scale, is recommended.

References

1. Breuer E, Lee L, De Silva M, Lund C. Using theory of change to design and evaluate public health interventions: a systematic review. Implement Sci. 2016;11(63):1–17.
2. Chan EY, Wu LT, Ng EJY, Glass GF Jr, Tan RHT. Applying the RE-AIM framework to evaluate a holistic caregiver-centric hospital-to-home programme: a feasibility study on Carer Matters. BMC Health Serv Res. 2022 Jul 19;22(1):933. doi: 10.1186/s12913-022-08317-3.
